# Rhabdomyolysis Caused by Isotretinoin and Exercise in an Otherwise Healthy Female Patient

**DOI:** 10.7759/cureus.25981

**Published:** 2022-06-15

**Authors:** Eli Raneses, Eric C Schmidgal

**Affiliations:** 1 General Medicine, Naval Hospital Camp Pendleton, Oceanside, USA; 2 Department of Dermatology, Naval Medical Center San Diego, San Diego, USA

**Keywords:** isotretinoin, scarring acne, nodulocystic acne, drug side effect, drug reaction, rhabdomyolysis, acne vulgaris

## Abstract

Acne vulgaris is one of the most common skin conditions treated by healthcare providers. Isotretinoin is a well-known and effective treatment for nodulocystic and scarring acne. Rarely, and usually in combination with exercise, patients treated with isotretinoin can develop rhabdomyolysis, a potentially life-threatening breakdown of muscle associated with elevated creatine kinase (CK). Here, we report a rare case of a female patient developing rhabdomyolysis three months after starting exercise and isotretinoin. She was treated with supportive care and medication was discontinued, resulting in a full recovery. Careful inquiry into the patient's exercise habits, along with a thorough review of systems at each visit can help identify high-risk patients. Routine monitoring of liver enzymes, specifically aspartate aminotransferase elevations, may provide a prompt to check a patient’s CK. Though regular monitoring of CK is not currently recommended, given the prevalence of regular exercise in certain patient populations, this case reinforces the importance of counseling patients on this potential side effect.

## Introduction

Acne vulgaris is one of the most common dermatologic conditions treated by healthcare providers, with an estimated prevalence of around 50 million individuals per year in the USA [1]. Isotretinoin, a systemic retinoid, is the only treatment with evidence of long-term remission of acne and is the treatment of choice for nodulocystic and recalcitrant scarring acne [2,3]. It is recognized that there can be a lag period of one to three months prior to observing a clinical effect. Systemic retinoids, though highly effective, have a number of documented adverse side effects, including cheilitis, xerosis with pruritus, teratogenicity, bone toxicity (such as diffuse idiopathic skeletal hyperostosis syndrome-like changes), hepatoxicity, psychiatric effects, and muscle pain [2,3]. Though muscle side effects are recognized, patients rarely develop rhabdomyolysis [4]. We present a case of an active duty military female patient with rhabdomyolysis following treatment with isotretinoin.

## Case presentation

A 26-year-old active-duty military female with no significant past medical history presented to the dermatology clinic to initiate further treatment for nodulocystic acne with scarring. She had a 13-year history of acne that had failed over-the-counter and prescription topicals, as well as oral antibiotics. Her only medication was a combined oral contraceptive (drospirenone/ethinyl estradiol). Her initial screening labs were within normal limits and her weight was 55kg. She was started on oral isotretinoin 40mg daily which was increased to 60mg (1.1mg/kg) daily after her first month of treatment. She tolerated this well and had no abnormalities on routine labs which included a complete blood count (CBC), complete metabolic panel (CMP), and lipids. The patient tolerated two months of treatment with reported side effects of dry skin and cheilitis.

At the start of the third month of treatment, and after reaching a cumulative isotretinoin dose of 3,000mg, the patient experienced an asymptomatic increase in her alanine aminotransferase (ALT) from 12U/L to 68U/L (Reference range: 17 - 63U/L) and aspartate aminotransferase (AST) of 17U/L to 74U/L (Reference range: 12 - 39 U/L). Aside from cheilitis, the patient was asymptomatic at this time, and this increase was found to be in the context of recently starting a branched-chain amino acid exercise supplement. The patient was advised to discontinue supplement use and treatment was continued. 

Through the third month of treatment, the patient noted vague myalgias without other symptoms and was found in routine labs to have worsening transaminitis with ALT 76U/L and AST 290U/L. The patient had discontinued supplement use by this time and denied any alcohol use. Given the reported myalgias and transaminitis, a creatine kinase (CK) was drawn and was significantly elevated to 25,521U/L (Reference range: 38 - 234 U/L). The patient was sent to the emergency department (ED) for acute evaluation. ED evaluation was significant for mild bilateral biceps tenderness and trace blood on urinalysis without evidence of kidney damage (serum creatinine within normal limits). It was noted at this time that the patient had started an exercise routine around the time of isotretinoin initiation three months prior. Her exercise routine was reported to be a combination of weight lifting and aerobic exercise (i.e. running) three times per week. She did not report recently increasing her workout intensity and she reported that her last workout was three days prior to ED presentation. The patient was given IV hydration, isotretinoin was discontinued and the patient was advised to temporarily discontinue exercising. The patient was discharged with close interval outpatient follow-up.

After isotretinoin cessation, increased hydration and stopping her workouts, the patient’s labs quickly down-trended to CK 3,129U/L over the next 3 days, with an associated decrease of ALT to 59U/L and AST to 81U/L. The patient’s CK completely normalized by 2 weeks, and she wished to restart isotretinoin. She agreed to decrease exercising while taking the medication. The patient was restarted at isotretinoin 20mg per day with close interval follow-up appointments and frequent lab monitoring. She tolerated the remainder of her isotretinoin course without subsequent lab abnormalities or symptoms and experienced complete clearance of her acne.

## Discussion

Rhabdomyolysis is a potentially life-threatening condition caused by the rapid breakdown of skeletal muscle fibers and the subsequent release of intracellular contents which can cause electrolyte disturbances and acute kidney injury (AKI) [5-7]. It is a clinical diagnosis with classic features such as muscle soreness, weakness, and myoglobinuria, with a CK that is generally elevated greater than five times the upper limit of normal and is often found to be greater than 1,000 U/L [8]. Around 26,000 cases of rhabdomyolysis are reported in the US annually, with a mortality rate reported from 10%, up to 22% in critical patients without AKI [5,7]. At least 1 fatal case of rhabdomyolysis has been associated with isotretinoin use [9].

The majority of patients who have been identified as having rhabdomyolysis associated with isotretinoin use have been male [10]. Transaminitis, specifically a significant elevation of AST, has been associated with rhabdomyolysis in the absence of any significant liver injury and, as with our patient, the elevated AST resolves as the CK decreases [11,12]. Previous reviews have identified elevations in CK in patients taking isotretinoin, though these elevations are generally mild, unlike this patient who experienced a CK increase of more than 100 times the upper limit of normal [10,13-15]. Sixty percent of rhabdomyolysis cases are found to have at least two contributing factors and CK elevations may synergistically increase in the setting of exercise and isotretinoin treatment [5,14]. This patient recovered after a period of discontinuation of medication and exercise and she was able to resume isotretinoin at a low dose without re-development of symptoms. This provides further evidence that exercise and isotretinoin may also act synergistically to cause rhabdomyolysis. The underlying etiology of isotretinoin increasing CK and causing rhabdomyolysis has not been fully elucidated, though at least one study points to isotretinoin-induced expression of apoptotic signaling and apoptotic proteins [16]. The first steps of treatment after patient stabilization and hydration are to stop isotretinoin and decrease or stop exercise if implicated, as in our patient. In the acute setting, patients require aggressive hydration and close monitoring, especially if their CK is severely elevated. Our patient did not have electrolyte disturbances or kidney injury, and she was able to be hydrated and discharged with close follow-up.

This case differs from most previous reports in that the patient was female, she had markedly elevated CK, and had been exercising for months prior to developing rhabdomyolysis [10]. Additionally, though single bouts of vigorous exercise have been implicated in the development of rhabdomyolysis, this case provides an example of rhabdomyolysis developing in a patient participating in consistent exercise without a discrete increase in intensity. Though this patient reported vague myalgia in the month prior to presentation, often patients may have mild symptoms, and as reported in small case series, patients who present with myalgia often do not have elevated CK [13,14]. This patient was also taking a workout supplement briefly while taking isotretinoin. This was not thought to contribute to the patient’s development of rhabdomyolysis as she was on it for a brief period, had discontinued it prior to rhabdomyolysis onset, and had worsening transaminitis despite discontinuation of the supplement. Our patient was taking around 1mg/kg/day isotretinoin for two months when she developed rhabdomyolysis. It has been reported that taking lower doses can be efficacious and have a lower incidence of severe side effects [17]. However, there has been a report of a patient that developed rhabdomyolysis on a low-dose regime as well [15]. Finally, our patient is an active duty military member. Military members may be required to participate in physical training and regular exercise, and there are policies in place to mitigate medication risks to service members, including from acne medications [18,19]. It may be important to counsel all patient's on the rare risk of rhabdomyolysis associated with isotretinoin and exercise. However, given this population’s potential regular exposure to exercise, special consideration should be taken when advising them of this side effect.

## Conclusions

Isotretinoin is a common and effective treatment for nodulocystic and recalcitrant acne vulgaris. This case presents a female patient with nodulocystic and recalcitrant acne treated with isotretinoin, who was exercising for several months without an increase in intensity, and developed rhabdomyolysis. Exercise and systemic isotretinoin treatment may act synergistically to cause rhabdomyolysis. Though rhabdomyolysis is a recognized potential adverse effect from isotretinoin use, routine monitoring or screening is not currently recommended. Given its rarity, routine laboratory monitoring is likely not needed. In the context of elevating liver enzymes, specifically AST, in patients taking isotretinoin, checking a CK may prove useful in identifying rhabdomyolysis, especially as many patients may initially experience mild or no symptoms. Awareness of this side effect and the association with exercise may lend itself to counseling patients on the risks associated with concomitant exercise. This recommendation may be especially relevant for certain patient populations (such as military members), who may engage in exercise more regularly than other populations, and who may also have mandatory physical training requirements.
